# Limb Remote Ischemic Conditioning Promotes Myelination by Upregulating PTEN/Akt/mTOR Signaling Activities after Chronic Cerebral Hypoperfusion

**DOI:** 10.14336/AD.2016.1227

**Published:** 2017-07-21

**Authors:** Xiaohua Li, Changhong Ren, Sijie Li, Rongrong Han, Jinhuan Gao, Qingjian Huang, Kunlin Jin, Yinghao Luo, Xunming Ji

**Affiliations:** ^1^Institute of Hypoxia Medicine, Xuanwu hospital, Capital Medical University, Beijing 100053, China; ^2^Department of China-America Institute of Neuroscience, Xuanwu Hospital, Capital Medical University, Beijing 100053, China; ^3^Cerebrovascular Diseases Research Institute, Xuanwu Hospital, Capital Medical University, Beijing, China; ^4^Center for Neuroscience Discovery, Institute for Healthy Aging, University of North Texas Health Science Center at Fort Worth, Texas 76107, USA; ^5^Emergency department, Xuanwu hospital, Capital Medical University, Beijing 100053, China; ^6^Beijing Key Laboratory of Hypoxia Translational Medicine, Beijing 100053, China

**Keywords:** cerebral hypoperfusion, white matter, vascular dementia, oligodendrocyte, ischemic conditioning, PI3K/Akt/mTOR

## Abstract

Limb Remote ischemic conditioning (LRIC) has been proved to be a promising neuroprotective method in white matter lesions after ischemia; however, its mechanism underlying protection after chronic cerebral hypoperfusion remains largely unknown. Here, we investigated whether LRIC promoted myelin growth by activating PI3K/Akt/mTOR signal pathway in a rat chronic hypoperfusion model. Thirty adult male Sprague Dawley underwent permanent double carotid artery (2VO), and limb remote ischemic conditioning was applied for 3 days after the 2VO surgery. Cognitive function, oligodendrocyte counts, myelin density, apoptosis and proliferation activity, as well as PTEN/Akt/mTOR signaling activity were determined 4 weeks after treatment. We found that LRIC significantly inhibited oligodendrocytes apoptosis (*p*<0.05), promoted myelination (*p*<0.01) in the corpus callosum and improved spatial learning impairment (*p*<0.05) at 4 weeks after chronic cerebral hypoperfusion. Oligodendrocytes proliferation, along with demyelination, in corpus callosum were not obviously affected by LRIC (*p*>0.05). Western blot analysis indicated that LRIC upregulated PTEN/Akt/mTOR signaling activities in corpus callosum (*p*<0.05). Our results suggest that LRIC exerts neuroprotective effect on white matter injuries through activating PTEN/Akt/mTOR signaling pathway after chronic cerebral hypoperfusion.

Vascular dementia is a heterogeneous group of brain disorders, in which cognitive impairment is attributable to cerebrovascular pathologies, and responsible for at least 20% of cases of dementia, being second only to Alzheimer’s disease (AD) [1]. Diffuse white matter lesions are essential pathological change of vascular dementia [2]. The primary cerebrovascular pathologies include multi-arteriolosclerosis, carotid stenosis or occlusion [3, 4], which lead to cerebral hypoperfusion, especially in deep white matter regions. White matter injuries, also termed leukoaraios, are very common in clinic.

White matter, comprises over half the human brain, is mainly composed of bundles of myelin-ensheathed axons, myelin-producing oligodendrocytes, astrocytes and microglia [5], and plays an essential role in signal transmission [6] and metabolic exchange [7, 8]. The pathogenic mechanisms responsible for hypoperfusion-mediated white matter lesions include blood-brain barrier (BBB) leakage [9, 10], inflammation [11, 12] and oxidative stress [13], which collectively lead to apoptosis of oligodendrocyte cells and demyelination. Demyelination or loss of sheath is a critical feature of white matter lesions induced by cerebral hypoperfusion [14].

Phosphatidylinositol 3-kinase/Akt (PI3K/Akt/mTOR) signaling is a serine/threonine kinase that regulates many intracellular molecules involved in basic processes [15, 16]. The effects of anti-apoptosis and promoting oligodendrocytes proliferation, increasing myelin gene expression lead PI3K/Akt/mTOR pathway as one of essential signaling pathways during myelin growth [17]. Several studies showed that PI3K/Akt/mTOR signalings involved in white matter injuries after chronic cerebral hypoperfusion in rats [18-20].

Limb remote ischemic conditioning (LRIC) is a phenomenon that brief and non-lethal blocking limbs blood flow can protect distant organs such as the brain, heart, and kidney from lethal ischemic injury [21]. Studies demonstrated the neuroprotective effect of LRIC in acute ischemic stroke via activating the PI3K/Akt/mTOR signal pathway [22, 23]. LRIC also protected whiter matter integrity after ischemia [24]. However, whether PI3K/Akt/mTOR signal pathway involves in the LRIC-mediated protective effect on white matter lesions after chronic cerebral hypoperfusion remains largely unknown. In the present study, we used a rat chronic cerebral hypoperfusion model to explore whether LRIC alleviates the loss of sheath by regulating PI3K/Akt/mTOR signal pathway.

## MATERIALS AND METHODS

### Animals

All animal experiments were approved by Animal Care and Use Committee of Xuanwu Hospital, Capital Medical University, China, and conducted according to the National Institutes of Health guidelines. Thirty adult male Sprague-Dawley rats (220 to 260 g weight) were purchased from Vital River Laboratories, Beijing, China, and maintained on a 12-hour light/dark cycle with unlimited access to food and water.

### Chronic Cerebral Hypoperfusion Model

Chronic cerebral hypoperfusion model was induced using the double carotid artery or 2-vessel occlusion (2VO) model as described previously [25, 26]. Briefly, rats were anesthetized with 4.0% enflurane and then maintained on 1.5-2.0%% enoflurane in 70% N_2_O and 30% O_2_ using a small-animal anesthesia system. Through a midline cervical incision, the bilateral common carotid arteries were carefully separated from the cervical sympathetic and vagal nerves, and then one of artery was doubly ligated with silk sutures. After ten minutes, the other carotid artery was also ligated. The incision was closed eventually. During the operation, rectal temperature was monitored and maintained at 37±0.5°C with heating blanket. The rats in sham group underwent an identical surgery except that the carotid arteries were not occluded.

### Limb remote ischemic conditioning

Rats (n=30) were randomly assigned to three groups: sham-operated group, 2VO group and 2VO + LRIC group. Each group included 10 animals. LRIC was initiated at 3 days after the hypoperfusion model by occluding blood flow to the hind limbs bilaterally while under anesthesia, once a day for 28 days. Hind limb occlusion was accomplished by tightening a tourniquet (8 mm) around the upper thigh for 3 cycles. For each cycle, the occlusion and release phase lasted 10 minutes, respectively. The rats in the sham and 2VO groups were only under anesthesia as the 2VO+ LRIC group.

### Morris water maze

The Morris Water Maze was carried out on the 21^th^ day after 3 weeks of LRIC to assess animal spatial learning and memory ability. The water maze used in our study was a flat black galvanized metal tank that was 210 cm in diameter and equipped with a platform 1–2 cm below the surface of the water. The rats were trained for 5 consecutive days, followed by the probe trial on day 6 with the original platform removed. The rats were trained four times per day (120 sec/trial), and were let down in four random places (N, W, SE, NW) in the pool. If the animals failed to locate the platform within 120 s, they were put on the platform and stayed for 20s before the next swim trial. The latency was recorded as 120 s. If the animals reached the platform within 120 s, they were immediately removed from the platform. On day 6, a spatial probe trial was conducted with the original platform removed to evaluate memory retention. The animals were let down diagonally from the platform. The cumulative time spending in target quadrant where the platform was located was recorded during a period of 60s. Finally, the platform was above the surface of water by discharging some water, and the rats were let down to find out the platform. The swimming distance, the time required to reach the platform and the swimming speed were recorded.

### Tissue collection and processing

At 4 weeks, all rats were treated with chloral hyrate (400 mg/kg, intraperitoneal) and perfused with saline. One half of brains were removed and post-fixed in 4% PFA for 24 hrs, and then stored in sucrose solution (30% in PBS) for 48 hrs at 4°C. Brains were placed into embedding medium for frozen tissue specimens, and then were stored at −80 °C. The other half was removed and put on the ice immediately. Then the corpus callosum was dissected carefully from these brains, and stored at -80°C.

### Immunofluorescence staining

Brain cryosections (10 µm) were put in sodium citrate buffer (10 mM, pH 6.0, 95°C; 10 min) for antigen retrieval. Sections were washed with PBS and then incubated in blocking solution (0.1% bovine serum albumin (BSA) for 1 hr. Sections were then incubated overnight at 4^?^C with primary antibody against MBP (1:1000, Abcam, USA), GST-pi (1:100, Abcam, USA) and Bromodeoxyuridine (1:500, Roche, USA). On the following day, after washing, the sections were subsequently incubated with immunofluorescence-conjugated IgG (1:500, Life Technologies, USA) for 2 h at room temperature. After washing, the sections were mounted on glass slides using fluorescent mounting medium with DAPI. The fluorescent signals were detected at excitation of 488 nm and 594 nm.

### Western blot

The white matter extracted from each brain was homogenized by ultrasonication in RIPA buffer containing a protease inhibitor cocktail. Protein concentrations were determined using the BCA method. Equal amounts of total protein (40-80µg) for each sample were loaded on 8-12% polyacrylamide gel and then transferred to PVDF membranes (Millipore). Membranes were blocked for 1 hr with 5% skim milk in Tris Buffered Saline with Tween (TBST) and soaked with primary antibodies against MBP (1:2000; Abcam), p-PTEN (1:1000; Cell Signaling Technology), Akt (1:1000; Cell Signaling), p-Akt (1:1000, Cell Signaling Technology), mTOR (1:1000, Cell Signaling, p-mTOR (1:1000, Cell Signaling Technology), GAPDH (1:1000, Zhongshanjinqiao), β-actin (1:1000, Zhongshanjinqiao) overnight at 4°C, respectively. Protein levels were expressed as the ratio to GAPDH or β-actin. Incubated membranes were then treated with secondary antibody conjugated with horseradish peroxidase in TBST for 1 hr at room temperature. The specific reaction was visualized by the chemiluminescence substrate luminol reagent (GE Healthcare, UK). The optical density of each resulting labeled band was measured in an image analysis program (Image proPlus 6.0).

### BrdU injections

To analyze cell renewal processes, from 14 days after LRIC, 5-Bromo-2′-deoxyuridine (BrdU, Sigma) was injected intraperitoneally to a dose of 50 mg/kg every second day for 2 weeks. Two extra steps included DNA denaturation (1 mM HCL at 37°C) for 30 min and HCl neutralization (sodium borate buffer, 50 mM, pH=8.5) for 20 min for BrdU staining.

**Figure 1.**
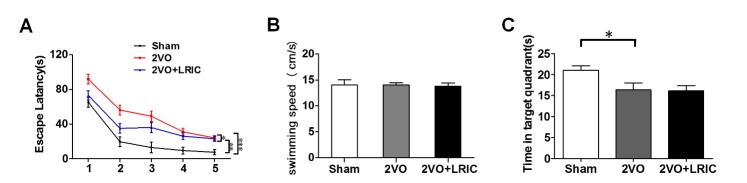
LRIC improves the chronic cerebral hypoperfusion-mediated spatial learning impairment Mean escape latency to the platform in the Morris water tes (**A**) t.*p<0.05: 2VO versus 2VO+LRIC group; **p<0.01: Sham versus 2VO+LRIC group; ***p<0.001: Sham versus 2VO group. Mean swimming speed in the Morris water maze (**B**). The mean time spent in target quadrant in probe test among different groups (**C**). Values are mean ± SEM. N=10/group. *p<0.05, **p<0.01, ***p<0.001.

### Cell death detection

*In Situ* Cell Death Detection Kit, POD (Tunel staining, Roche, USA) was used to analyze the apoptosis of oligodendrocytes according to the manufacturer’s instructions.

### Statistical analysis

All data is expressed as mean ± standard error of the mean (SEM). SPSS 16.0 was used to perform repeated measures ANOVA to analysis the escape latency. One-way ANOVA with Tukey’s post hoc multiple comparison test was used to determine any significant difference between the different treatment groups for other parameters. A value of *p*<0.05 was considered statistically significant.

## RESULTS

### LRIC improves the chronic cerebral hypoperfusion-mediated spatial learning impairment

**Figure 2.**
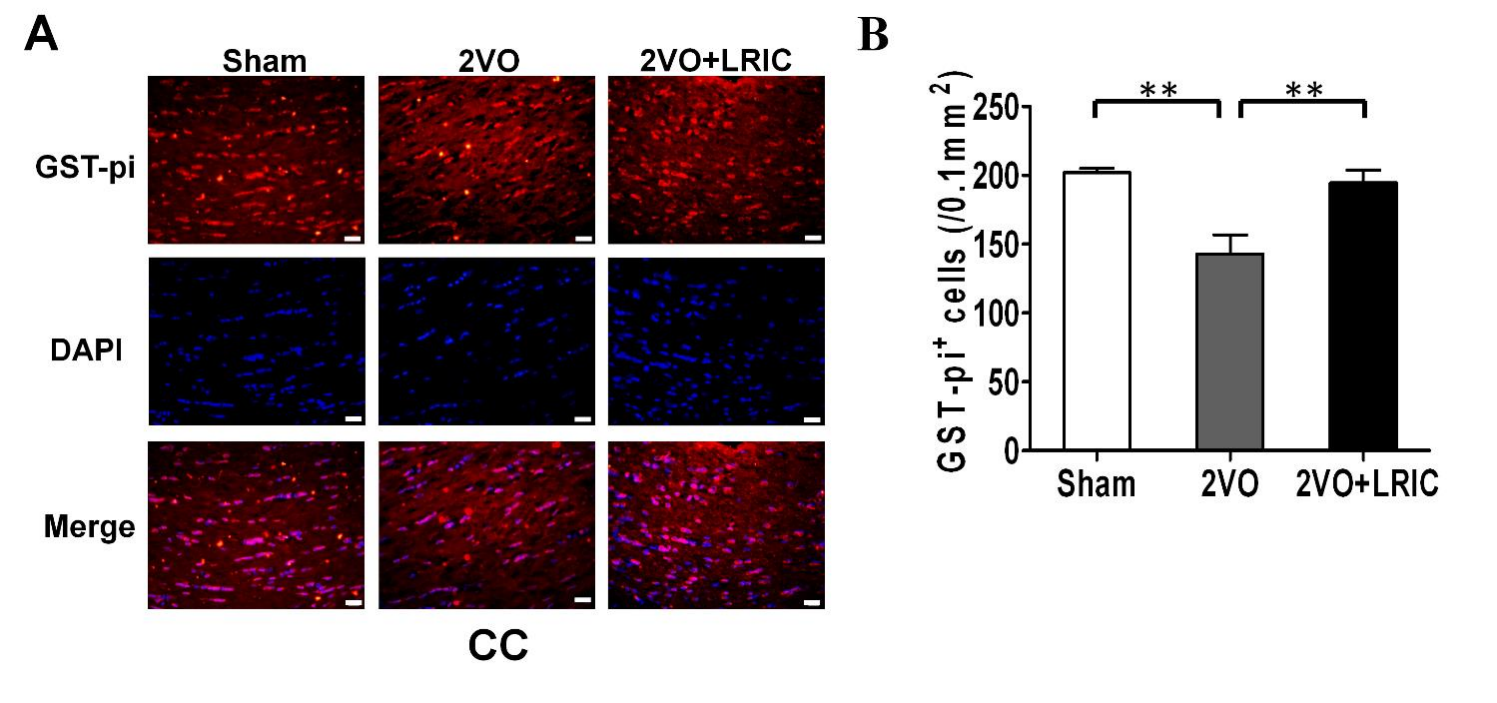
LRIC reduces the loss of oligodendrocytes in corpus callosum after chronic cerebral hypoperfusion (**A**) Representative images of GST-pi positive cells in corpus callosum at day of 30. GST-pi: red; DAPI: blue. Scale bar: 20μm. (**B**) Numbers of GST-pi-positive (GST-pi+) cells in corpus callosum at day 30. Values are expressed as mean ± SEM. N = 5/group. *p<0.05, **p<0.01, ***p<0.001.

Morris Water Maze test was used to test the effect of LRIC on cognitive impairments after chronic cerebral hypoperfusion. The escape latency (time required to reach the platform) was used to assess learning capacity. We found that there was a significant difference in escape latency (F (2, 27) =17.1, *p*?<?0.01, Fig. 1A) among the Sham, 2VO and 2VO+LRIC group. The mean escape latency over a period of 5 days for the 2VO group (49.9±3.2 s) and 2VO+LRIC group (39.2±3.2 s) were significantly longer than Sham group (22.9±3.3 s). While the mean escape latency of 2VO+LRIC group was shorter than 2VO group (*p*<?0.05). In the first two days, the escape latency in 2VO+LRIC group was significantly shorter than 2VO group (*p*<0.05), then the time required to reach the platform became eventually equal in the following three days. There was no difference in swimming speed among different groups (*p*>0.05, Fig. 1B) in last exposed platform test, which suggested the motor function was consistent among three groups. In probe trial on day 6, the rats in sham group spent more time in target quadrant (21.0±1.0s) than the animals in 2VO (16.3±1.6s, *p*<0.05, Fig. 1C) and 2VO+LRIC group (16.1±1.3s, *p*<0.05, Fig. 1C). But the time spent in target quadrant in 2VO group was not different from 2VO+LRIC group (*p*>0.05*,* Fig. 1C).

### LRIC reduces the loss of oligodendrocytes and demyelination in corpus callosum after chronic cerebral hypoperfusion

**Figure 3.**
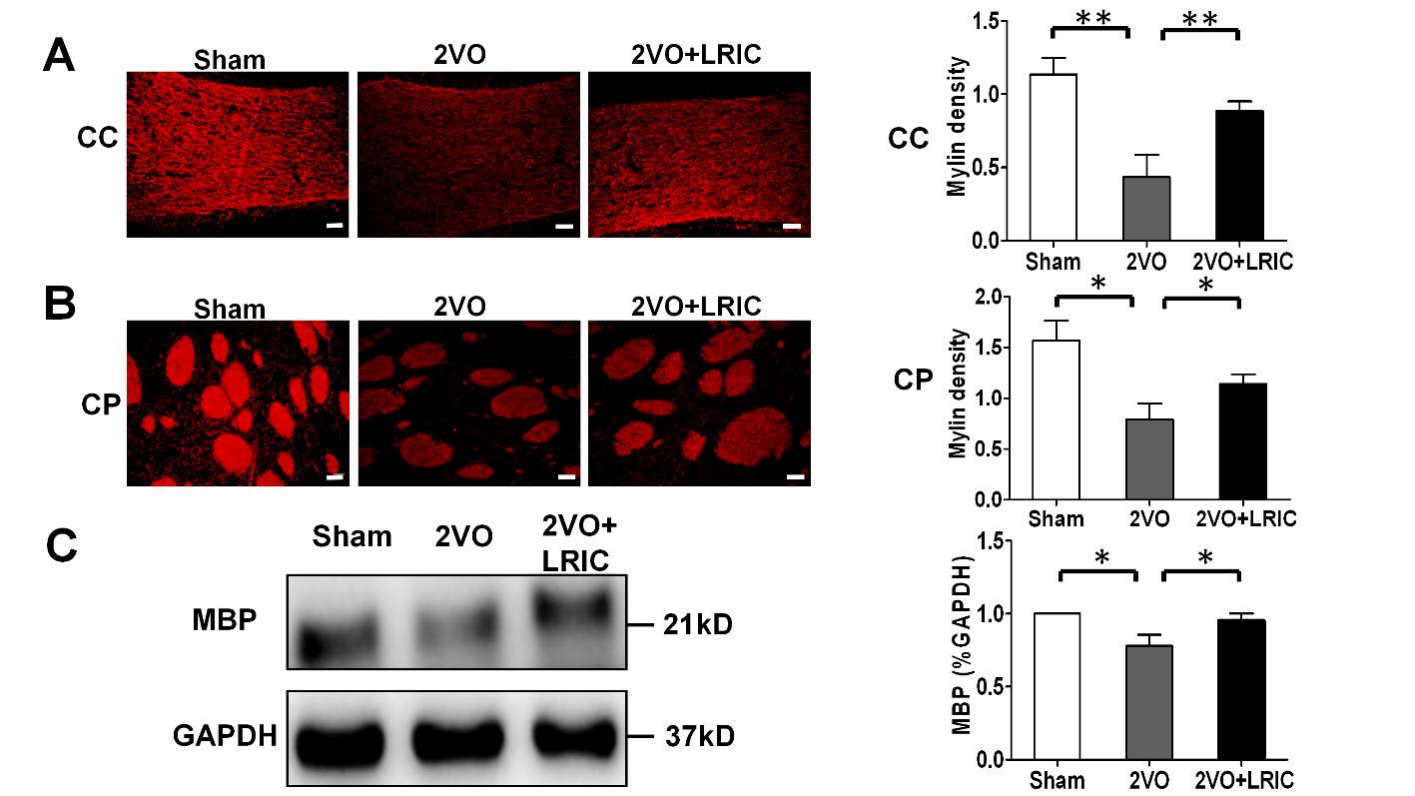
LRIC promotes myelination in corpus callosum after chronic hypoperfusion Representative images (left panel) and quantitative (right panel) of MBP staining among different groups (**A, B**). Scale bar, 20 μm Values are mean ± SEM. N = 5 per group. Representative Western blots (left panel) and quantitative analysis (right panel) of MBP expression at day 30 in corpus callosum (**C**). CC: corpus callosum; CP: caudate putamen. Values are mean ± SEM. N = 4 per group. *p<0.05, **p<0.01, ***p<0.001.

For 4 weeks after onset of cerebral hypoperfusion, the number of oligodendrocytes in corpus callosum was assessed with fluorescent staining against GST-pi. Compared to sham group, the GST-pi-positive cells were substantially decreased in corpus callosum after 2VO model (*p*<0.01, Fig. 2A and 2B). However, LRIC significantly increased the number of GST-pi^+^cells in corpus callosum (*p*<0.01, Fig. 2A and 2B). As expected, after cerebral hypoperfusion, the corpus callosum (*p*<0.01, Fig. 3A) and caudate putamen (*p*<0.05, Figure 3B) regions showed a reduction in myelin staining and decreased myelin basic protein expression in Western blots (*p*<0.05, Fig. 3C). LRIC also significantly increased myelin staining (*p*<0.01, Fig. 3A and 3B) and myelin basic protein expression (*p*<0.05, Fig. 3C).

### LRIC decreases oligodendrocytes apoptosis after chronic hypoperfusion

**Figure 4.**
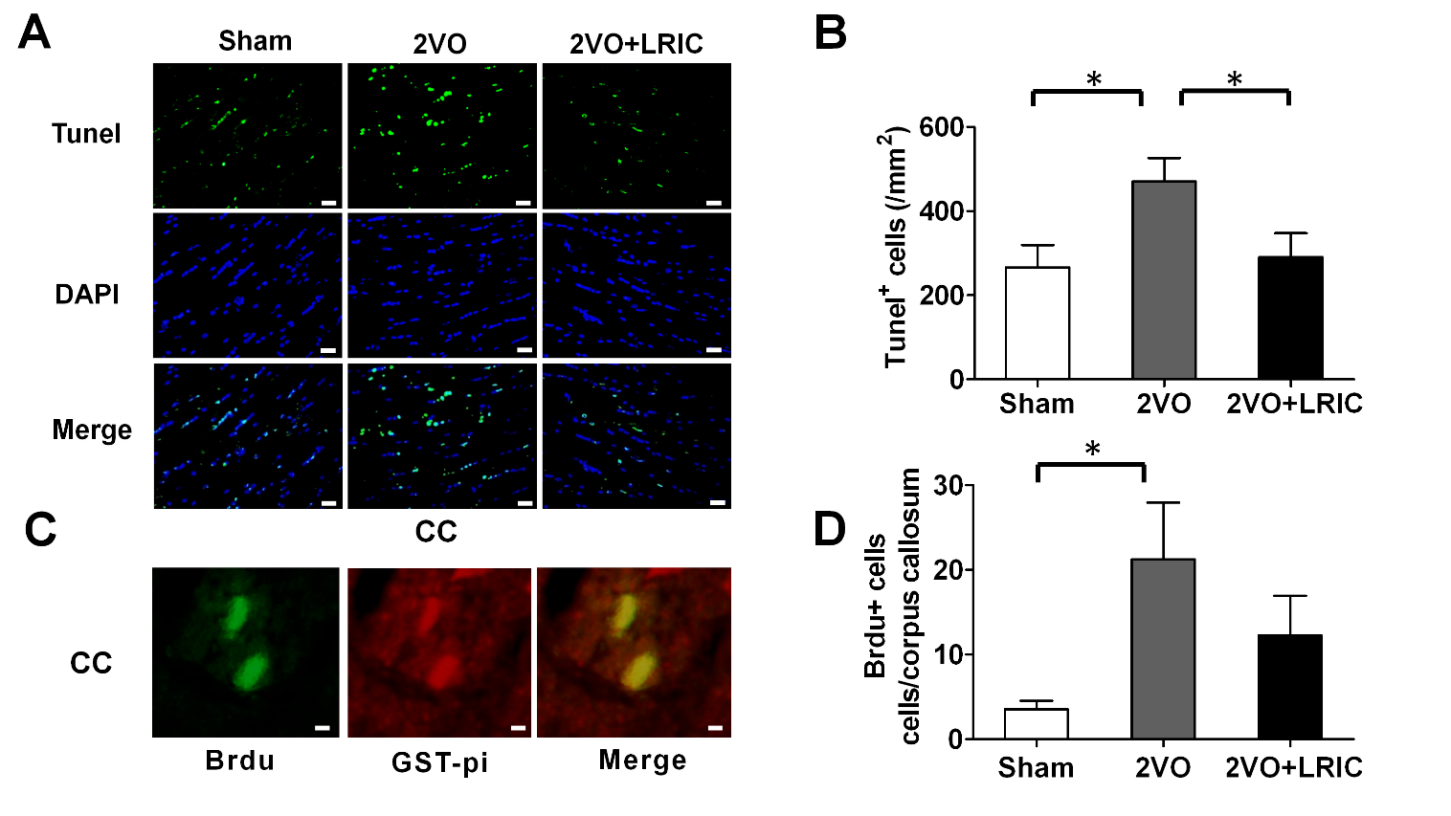
LRIC decreases oligodendrocytes apoptosis after chronic hypoperfusion Representative images of Tunel-positive cells in corpus callosum at day of 30 (**A**). Tunel: green; DAPI: blue. Scale bar: 20 μm. Quantification Tunel-positive cells in the corpus callosum (**B**). Representative images double staining of Brdu with GST-pi in corpus callosum at day of 30 (**C**). Brdu: green; GST-pi: red. Scale bar: 20 μm. Quantification Brdu+ cells in the corpus callosum (**D**). Values are mean ± SEM (N=5). *p<0.05.

To identify whether the effect of LRIC on sustaining oligodendrocyte viability is attributed to decreasing oligodendrocytes apoptosis or increasing its proliferation, we performed TUNEL and BrdU staining respectively. The double staining showed that chronic cerebral hypoperfusion conduced severe oligodendrocytes apoptosis in corpus callosum compared with sham group (*p*<0.05, Fig. 4A and 4B); while LRIC obviously reduced oligodendrocytes apoptosis (*p*<0.05, Fig. 4A and 4B). The BrdU staining revealed that the renewal process was activated along with the white matter injury after chronic cerebral hypoperfusion, as in 2VO group, the number of BrdU incorporated oligodendrocyte cells in corpus callosum was significantly increased compared with sham group (*p*<0.05, Fig. 4C and 4D). But no difference was found in the number of BrdU-positive cells in corpus callosum between 2VO and 2VO +LRIC groups (*p*>0.05, Fig. 4C and 4D).

### LRIC activated the PI3K/Akt/mTOR signaling in corpus callosum

**Figure 5.**
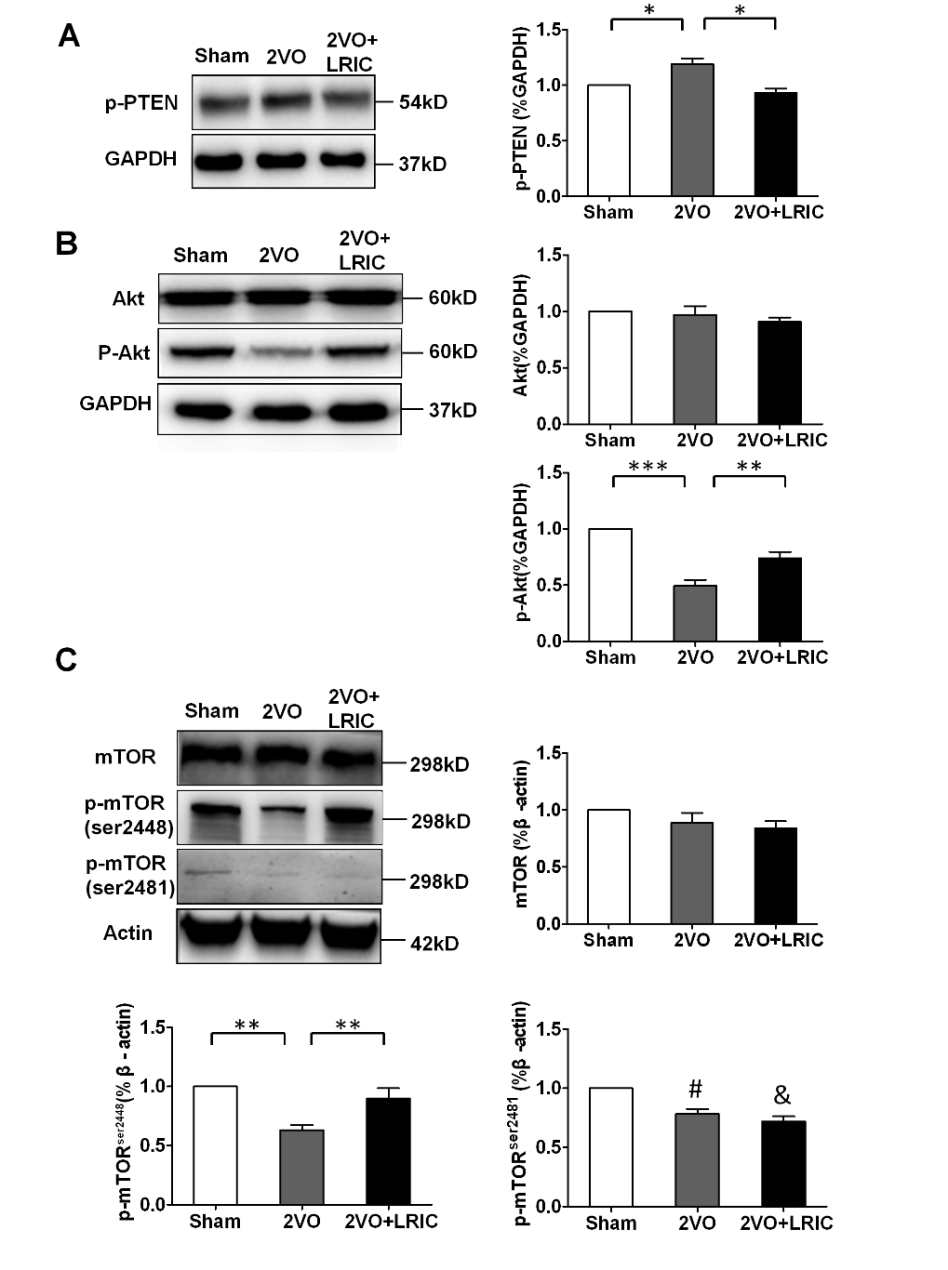
LRIC activated the PI3K/Akt/mTOR signaling in corpus callosum Representative Western blots (left panel) and quantitative analysis (right panel) of p-PTEN, Akt and p-Akt, mTOR, p-mTOR^ser2448^and p-mTOR^ser2481^ protein levels in different groups (**A, B, C**). Data are presented as mean ± SEM (N = 4 per group). ^#^ (*p*<0.01): 2VO versus sham group; ^&^ (*p*<0.01): 2VO+LRIC group versus sham group; *p<0.05, **p<0.01.

To investigate whether the PI3K/Akt/mTOR pathway was the intracellular mechanism underlying LRIC-mediared protective effect on demyelination, we first detected the expression of phosphorylated Phosphatase and tensin homologue (p-PTEN), as an upstream inhibitor, which downregulates the Akt activity. We found that the expression of p-PTEN was increased in corpus callosum after chronic cerebral hypoperfusion compared with sham group (*p*<0.05, Fig. 5A). However, after LRIC treatment, the level of p-PTEN was significantly decreased compared with 2VO group (*p*<0.01, Fig. 5A). Consistently, the expression of p-Akt was obviously downregulated after chronic cerebral hypoperfusion compared with sham group (*p*<0.001, Fig. 5B). However, LRIC increased the level of p-Akt (*p*<0.01, Fig. 5B). mTOR, as the AKT signaling downstream effector, exists in two functionally distinct complexes, the mTORC1 (raptor-mTOR complex) and the mTORC2 (rictor-mTOR complex), which were activated by phosphorylating on ser ^2448^ and ser^ 2481^ respectively. As the changes of p-Akt, the expression of p-mTOR ser ^2448^ was upregulated again by LRIC (*p*<0.01, Fig. 5C). After hypoperfusion, the expression of mTOR ser^2481^ was markedly decreased in 2VO and 2VO +LRIC group compared with sham group (*p*<0.05, Fig. 5C). However, no difference was found between 2VO and 2VO + LRIC groups (*p*>0.05, Fig. 5C).

## DISCUSSION

In this study, we found that LRIC reduced oligodendrocytes apoptosis, alleviated demyelination in corpus callosum, and improved spatial learning performance after chronic cerebral hypoperfusion in rats. In addition, we also found that p-Akt and p-mTOR ser ^2448^ was obviously up-regulated after LRIC treatment. Our findings suggest that LRIC attenuated demyelination partially via activating PTEN/Akt/mTOR1 pathway.

Morris Water Maze is a standard method for spatial learning and memory, and they were assessed by the escape latency and the time spent in target quadrant separately [27]. Our results showed that the escape latency in 2VO +LRIC group was significantly shorter than 2VO group, so we considered that the LRIC-treated rats learned more quickly. But there was no difference in the time spent in target quadrant between 2VO+ LRIC and 2VO group. From a pathological perspective, myelin is a cholesterol rich extension of oligodendrocytes plasma membrane [28], which sharply facilitates axon signal conduction [6]. And optimizing the speed of impulse transmission is an important factor for learning. So, myelin mainly participates during leaning [29, 30]. While memory process mainly dependents on hippocampus and frontal cortex [31]. Demyelination or the loss of the myelin sheath around axons could result in learning impairment [32]. Our results demonstrated that LRIC obviously alleviated demyelination and then improved spatial learning impairment after chronic cerebral hypoperfusion. Therefore, we speculated that LRIC improved the chronic cerebral hypoperfusion-mediated spatial learning impairment by promoting myelin repair.

Myelin is produced by oligodendrocytes in the central nervous system [33]. The apoptosis of oligodendrocyte cells after ischemia results in demyelination. Along with the demyelination, oligodendrocytes proliferation was activated to repair the myelin damage [34]. In this study, the oligodendrocytes proliferation was also observed after 28 days of 2VO. Our results showed that LRIC significantly increased the number of oligodendrocyte in corpus callosum after chronic cerebral hypoperfusion with GST-pi staining. Then we wondered whether the apoptosis of oligodendrocytes or the proliferation was involved into the LRIC-mediated protective effect on white matter. We found that LRIC decreased the apoptosis of oligodendrocytes but did not promote its proliferation after chronic cerebral hypoperfusion.

Multiple intracellular signaling pathways are responsible for the myelin growth. AKT, is a serine/threonine kinase that regulates many intracellular molecules involved in basic processes including cell growth, proliferation, and survival [15, 16]. mTOR is the core kinase in two structurally and functionally distinct complexes, mTORC1 and mTORC2. Several studied demonstrated that up-regulated AKT signaling increased the thickness of myelin [35, 36]. Accompanied the increased AKT signaling, the activity of mTORC1 also up-regulated [37]. Therefore, the PI3K/Akt/mTOR pathway is an important signaling pathways during oligodendrocytes survival and myelin growth. Several reports demonstrated that the PI3K/Akt/mTOR signal pathway were implicated into the protective effect on acute ischemic stroke mediated by LRIC [22, 23]. However, whether LRIC also activates PI3K/Akt/mTOR signal pathway in white matter after chronic cerebral hypoperfusion is still unknown. Our results showed that LRIC downregulated the expression of PTEN, upregulated the expression of p-Akt and p-mTOR, and finally decreased the loss of sheath. Thus, this is the first report to show that LRIC activated the PTEN/Akt/mTOR signaling pathway in white matter of the rat with chronic hypoperfusion model.

Limb remote ischemic conditioning (LRIC) is an intrinsic process in which shorter and non-lethal blocking limbs blood flow protects distant organs against the subsequent sustained lethal ischemic injury [21]. Yet, how the protective effect transmitted and communicated from the distant ischemic-reperfusion muscle to the brain remains a conundrum. Up to now, three potential mechanism have been proposed: (1) Humoral factors released in the pre-conditioned organ are transported via the blood circulation to protect the target organ [38]; (2) Neurogenic transmission with involvement of muscle afferents and the autonomic nervous system [38] and (3) Immunomodulation [39]. However, these proposed theories remain to be clarified. So far, several studies indicated that the underlying protective mechanisms of remote ischemic conditioning are associated with its ability to attenuate production of free radicals [40], promote the cell survival pathway [41], modulate the immune system [39] or to inhibit the apoptotic cell signaling pathways [42]. In addition, vast of the data have demonstrated that LRIC exerted protective effect on acute ischemia stroke [43], symptomatic intracranial atherosclerosis [44] and chronic cerebral ischemia [24]. Our study also demonstrated that LRIC protect white matter lesions after chronic cerebral hypoperfusion by activing PTEN/Akt/mTOR signaling pathway. In addition, LRIC is feasible and low cost. So LRIC represents a highly practical and translatable therapy for acute, subacute, and chronic neurological diseases.

In conclusion, we determined that LRIC decreased oligodendrocytes apoptosis, reduced the loss of sheath, and further improved the spatial learning performance after chronic cerebral hypoperfusion. Although the mechanisms involved in LRIC need to be further examined, the results from our study and the current literature supports the idea that the PTEN/Akt/mTOR are partially involved in the myelination induced by LRIC. The results demonstrated in this study may provide clues to help elucidate the protective capabilities of LRIC on white matter injury.
